# Corn Meal a Vehicle for Heat

**Published:** 1874-02

**Authors:** E. Cutter


*BOSTON JOURNAL OF CHEMISTRY--January,* 1874:

INDIAN MEAL A VEHICLE FOR HEAT.—*By E. Cutter.*—The application of heat to the body as a remedy is very common. Wringing out cloths in hot water, infusions of hops or other plants, in placing them upon the part affected, is probably the plan most frequently pursued. This practice is objectionable, because it needs close care to prevent wetting and chilling when cool, and also to be so frequently renewed and reapplied, to keep up the due amount of heat. When, therefore, it is desired to apply dry heat to any person, it is only required to place a quantity of the Indian meal in a baking pan on a heated stove, and stir constantly till thoroughly warmed. It should not be burnt. It can now be put into woolen sacks and tied up and applied as a hot bottle usually is, or into large flannel bags, if for the abdomen. In a case of successful resuscitation of new-born child, the heated meal was poured into an oblong chopping-tray, a flannel cloth laid over it, and the infant in it. The cloth yielded, and the child was partly buried in the warm meal. It is found that the meal retains its heat long, and when it cools it does not chill, which is a very important consideration. Two sets of bags or wrappers may be provided, so that while one is being applied the other may be heated. The meal is not weighty. The aroma of it when heated is rather agreeable than otherwise. It is now my favorite vehicle for applying heat.

